# How anthropogenic changes may affect soil-borne parasite diversity? Plant-parasitic nematode communities associated with olive trees in Morocco as a case study

**DOI:** 10.1186/s12898-016-0113-9

**Published:** 2017-02-06

**Authors:** Nadine Ali, Johannes Tavoillot, Guillaume Besnard, Bouchaib Khadari, Ewa Dmowska, Grażyna Winiszewska, Odile Fossati-Gaschignard, Mohammed Ater, Mohamed Aït Hamza, Abdelhamid El Mousadik, Aïcha El Oualkadi, Abdelmajid Moukhli, Laila Essalouh, Ahmed El Bakkali, Elodie Chapuis, Thierry Mateille

**Affiliations:** 1 Plant Protection Department, Faculty of Agriculture, Tishreen University, PO Box 2233, Latakia, Syrian Arab Republic; 2 IRD, UMR CBGP, 755 Avenue du Campus Agropolis, CS30016, 34988 Montferrier-sur-Lez Cedex, France; 3 CNRS, UMR EDB, Université Toulouse III Paul Sabatier, Bâtiment 4R1, 118 Route de Narbonne, 31062 Toulouse Cedex 9, France; 4 UMR AGAP, SUPAGRO, Campus CIRAD, TAA-108/03, Avenue Agropolis, 34398 Montpellier Cedex 5, France; 5 Museum and Institute of Zoology PAS, Wilcza 64, 00-679 Warsaw, Poland; 6 Faculté des Sciences et Techniques, Université Abdelmalek Essaadi, BP 2062, 93030 Tétouan, Morocco; 7 Laboratoire LBVRN, Faculté des Sciences d’Agadir, Université Ibn Zohr, BP 8106, 80000 Agadir, Morocco; 8 INRA, CRRA, BP 513, 40000 Marrakech, Morocco; 9 INRA, UMR APCRPG, BP 578, 50000 Meknes, Morocco; 10 IRD, UMR IPME (IRD/Université de Montpellier/CIRAD), 911 Avenue Agropolis, BP 64501, 34394 Montpellier Cedex 5, France; 11 UMR PVBMT, 3P-CIRAD, 7 chemin de l’Irat, Ligne paradis, 97410 Saint Pierre, Réunion

**Keywords:** Anthropisation, Communities, Functional diversity, Morocco, Olive, Plant-parasitic nematodes, Taxonomical structures

## Abstract

**Background:**

Plant-parasitic nematodes (PPN) are major crop pests. On olive (*Olea europaea*), they significantly contribute to economic losses in the top-ten olive producing countries in the world especially in nurseries and under cropping intensification. The diversity and the structure of PPN communities respond to environmental and anthropogenic forces. The olive tree is a good host plant model to understand the impact of such forces on PPN diversity since it grows according to different modalities (wild, feral and cultivated olives). A wide soil survey was conducted in several olive-growing regions in Morocco. The taxonomical and the functional diversity as well as the structures of PPN communities were described and then compared between non-cultivated (wild and feral forms) and cultivated (traditional and high-density olive cultivation) olives.

**Results:**

A high diversity of PPN with the detection of 117 species and 47 genera was revealed. Some taxa were recorded for the first time on olive trees worldwide and new species were also identified. Anthropogenic factors (wild vs cultivated conditions) strongly impacted the PPN diversity and the functional composition of communities because the species richness, the local diversity and the evenness of communities significantly decreased and the abundance of nematodes significantly increased in high-density conditions. Furthermore, these conditions exhibited many more obligate and colonizer PPN and less persister PPN compared to non-cultivated conditions. Taxonomical structures of communities were also impacted: genera such as *Xiphinema* spp. and *Heterodera* spp. were dominant in wild olive, whereas harmful taxa such as *Meloidogyne* spp. were especially enhanced in high-density orchards.

**Conclusions:**

Olive anthropogenic practices reduce the PPN diversity in communities and lead to changes of the community structures with the development of some damaging nematodes. The study underlined the PPN diversity as a relevant indicator to assess community pathogenicity. That could be taken into account in order to design control strategies based on community rearrangements and interactions between species instead of reducing the most pathogenic species.

## Background

A biological community refers to an assemblage of populations from different organisms living together in a habitat. This biological assemblage within a community could be described by several traits such as the number of species (richness), their relative abundance (evenness), the present species (taxonomical structure), the interactions among them as well as their temporal and spatial variation [1]. Species diversity is important for the stability of the community and consequently that of the ecosystems [2]. For instance, functional consequences on ecosystem processes are related to species richness and to species-specific traits. Moreover, species diversity can play a crucial role in ecosystems resilience and/or resistance to human disturbances and to environmental changes [1].

Soil communities have been described as the “poor man’s tropical rainforest”, because of the relatively high level of biodiversity and the large proportion of undescribed species, as well as the limited information available about their community structure and dynamics [3]. Human interventions in ecosystems such as land-use changes, invasive species and over-exploitation, lead to biodiversity loss and/or species extinction [4]. For example, in agrosystems, crop intensification greatly disturbs the soils, affecting composition and functions of their biota [5, 6].

Among soil biota, nematodes are ubiquitous soil inhabitants and among the most abundant and diversified biota [7]. They reflect several feeding behaviors that make it possible to allocate them to different trophic groups: bacterivores, fungivores, carnivores and plant feeders [8]. Due to the various life strategies of nematodes (*r* and *K* for colonizer and persister nematodes, respectively), their diversity and their co-existence in communities are closely related to short response time, to environmental changes and to disturbances in their habitats [9].

Plant-parasitic nematodes (PPN) are known to attack a wide range of crop plants (cereals, vegetables, tubers, fruits, flowers, etc.), causing annual crop losses estimated at billions of dollars in worldwide [10, 11]. On the olive tree (*Olea europaea* L.), PPN are able to reduce tree growth [12] and may be responsible for 5–10% yield losses [13]. Their impact is especially strengthened in nurseries and in intensive cultivation systems where irrigation conditions favor the development of roots and, as a result, nematode multiplication [14]. A high diversity of PPN on olive trees was reviewed worldwide [14, 15].

In Morocco, olive tree is a good example of ecological, botanical and genetic diversity. Spontaneous trees are distinguished under three different forms: (i) autochthonous wild trees, usually referred to as oleasters (*O. europaea* subsp. *europaea* var. *sylvestris* (Mill.) Lehr.), are common in coastal and mountainous regions [16]; (ii) the Moroccan hexaploid olive subspecies *O. europaea* subsp. *maroccana* is endemic in the High Atlas Mountains [17]; (iii) feral forms are wild-looking olive trees that correspond either to abandoned cultivated olive trees or to olive trees grown from cultivated olive seeds spread by birds. Additionally, cultivated forms (*O. europaea* subsp. *europaea* var. *europaea*) are also widespread. Different olive cropping systems can be distinguished according to tree density [18]: traditional orchards (ca. 80–400 trees/ha) vs high-density orchards (up to 1800 trees/ha). However, these new intensive techniques, accompanied by the replacement of traditional low-intensive production with highly intensified and mechanized cultivation, including the use of herbicides to remove weeds, are expected to induce a possible degradation of the plant communities and their associated fauna [19]. As for olive propagation, it is generally performed from root cuttings that could be accompanied by soil transport and, consequently, by the spread of soil-borne parasites. Thus, PPN could be spread by soil transport or by unsanitized plant material (e.g. from uncertified nurseries). The local PPN populations in olive-growing areas could therefore have originated from historical mixtures made up of native (before olive introduction) and invasive (with root stocks from oleasters) communities. In this context, we hypothesize that PPN communities may have adapted to olive propagation processes and to cultivation practices. These anthropogenic forces could exist in Morocco where high-density cultivated areas have been extended and where ancestral or traditional cultivars have often been discarded in favor of a few highly productive varieties [20]. These new conditions of cultivation might have to face a resurgence of several pests, including PPN. To address these hypotheses, this study was undertaken in order to: (i) describe the species diversity of PPN communities associated with wild, feral and cultivated olives in Morocco where their diversity is completely unknown, and (ii) assess how anthropogenic forces (propagation and intensification practices) could impact the diversity and the structure of PPN communities by comparing them between different olive growing modalities.

## Methods

### Site description

**Fig. 1:**
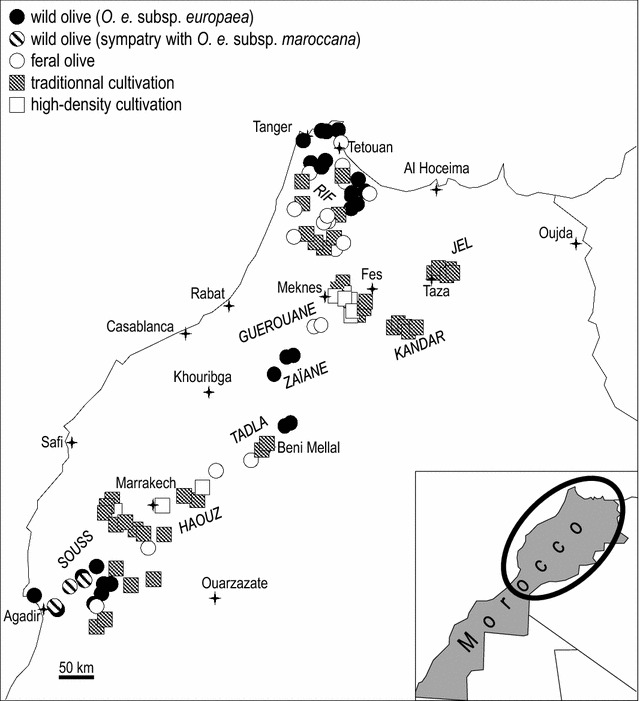
Sites sampled in Morocco. Olive-growing modalities are given for each site

Sampling of soil and olive leaves took place in Morocco from March to April 2012. Wild olive locations were as far as possible from current orchards. In contrast, feral olive locations were sampled within the proximity of cultivated olive stands or near main roads. The survey was conducted at 94 sites in several geographic regions all along a northeast-southwest 900-km long transect (Fig. 1; Table 1). The main regions sampled included: (i) the Souss region (15 sites), located on the southern side of the High Atlas Mountains near Agadir, where sampled trees were either wild (including trees of *O. europaea maroccana* in sympatry with *O. europaea* var. *sylvestris*), feral, or traditionally cultivated; (ii) the Haouz region (15 sites) located on the northern side of the High Atlas Mountains near Marrakech, where sampled trees were traditionally or high-density cultivated, or feral; (iii) the Tadla region (five sites) located along the northern side of the southern Middle Atlas Mountains near Beni Mellal, where sampled trees were either wild, feral, or traditionally cultivated; (iv) the Zaïane region (three wild olive sites), south of Meknes; (v) the Guerouane region (with traditionally or high-density cultivated sampled trees, and less feral trees); (vi) the Kandar region (five sites) located in the northern Middle Atlas Mountains, south of Fes and the Jel plain situated to the east of Taza in eastern Morocco (five sites), where trees are traditionally cultivated; and (vii) both the Atlantic and Mediterranean slopes of the Rif mountains in the north (33 sites) where most of the sampled trees were wild or feral, and less traditionally cultivated.

**Table 1:** Location of the olive sampling sites surveyed in Morocco

Geographic region	City	Olive modality	No of sites	Latitude N	Longitude W
				(decimal°)	(decimal°)
Souss	Tiguert	Wild	2	30.63	9.86
	Aourir	Wild	1	30.52	9.59
	Ouled Teïma	Wild	1	30.81	9.14
		Feral	1	30.42	9.02
		Traditional cultivation	1	30.42	9.02
	Taroudant	Wild	4	30.74	8.77
		Traditional cultivation	2	30.61	9.34
	Ouled Berhil	Traditional cultivation	1	30.65	8.18
	Aoulouz	Traditional cultivation	1	30.55	8.66
Haouz	El Kelaa Des Sraghna	Feral	1	32.15	7.26
		Traditional cultivation	1	31.37	7.95
	Tamellalt	Traditional cultivation	1	31.46	7.98
	Sidi Bou Othmane	High-density cultivation	1	31.70	7.69
	Marrakech	High-density cultivation	2	31.69	8.11
		Traditional cultivation	7	31.63	8.10
	Tahannaout	Traditional cultivation	1	31.57	7.97
	Asni	Feral	1	31.28	7.96
Tadla	Beni Mellal	Wild	2	32.58	5.98
		Traditional cultivation	1	32.21	6.83
	El Ksiba	Feral	2	32.32	6.39
Zaïane	Oulmes	Wild	2	33.32	6.07
	Oued Zem	Wild	1	33.33	6.00
Guerouane	El Hajeb	High-density cultivation	2	33.70	5.63
		Traditional cultivation	2	33.77	5.71
	Meknes	High-density cultivation	4	33.88	5.41
		Traditional cultivation	3	33.85	5.39
	Khemisset	Feral	2	33.63	5.83
Kandar	Sefrou	Traditional cultivation	5	33.87	4.88
Jel	Taza	Traditional cultivation	3	34.25	3.80
	Msoun	Traditional cultivation	2	34.26	3.74
Rif	Tanger	Wild	1	35.79	5.92
	Fnideq	Wild	2	35.78	5.37
	Tetouan	Wild	1	35.54	5.62
		Feral	1	34.79	5.77
	Asilah	Wild	4	35.07	5.33
		Traditional cultivation	1	35.05	5.35
	Chefchaouen	Wild	8	35.07	5.33
		Feral	3	35.07	5.32
		Traditional cultivation	2	35.38	5.37
	Bni Harchen	Wild	2	35.54	5.62
	Ouazzane	Wild	1	34.94	5.53
		Feral	2	34.79	5.77
		Traditional cultivation	5	34.79	5.77

### Soil sampling

Considering that PPN spend all or almost all their life in the soil [21], the nematode sampling only included soil. A total of 213 samples were collected from the 94 sites. This was done with a small spade under the foliage of each olive tree from the upper rhizosphere (the 15–20-cm deep layer inhabited by pleiotropic roots), in the close vicinity of active olive roots. This ensured that roots from weeds or other herbaceous plants were unlikely sampled. On cultivated olive (traditional and high-density cultivation), tillage and other human activities are frequent, which could lead to the homogenization of the PPN communities in an orchard. Each orchard was therefore considered as a repetition per modality. The sampling was carried out in each orchard along transects under four trees located at a distance of approximately 10 m. Five sub-samples were collected from each tree. These 20 sub-samples were thoroughly mixed to obtain a single representative sample per orchard. Contrary to cultivated orchards, heterogeneous PPN communities were expected in wild and feral olive trees because human interventions are scarce or absent. Each tree was thus taken as a repetition. Five sub-samples were also collected from each tree and then combined to form one 1-dm^3^ reference sample per tree.

### Genetic characterization of the olive tree

In order to confirm the determination of olive-growing modalities, three olive branches corresponding to soil samples were collected to determine the chloroplast haplotype of each tree (according to [22]). All cultivated olive sampled trees only show the haplotype E1-1. Feral olive sampled trees show only E1-1 or mixtures with E2 and E3 haplotypes (i.e., E2-1, E2-2, E2-4 and E3-3, E3-4). E2 and E3 have been previously detected in Moroccan cultivars, but with frequencies below 5% [16]. Wild sampled trees show haplotypes characteristic of Moroccan-Iberian oleasters (i.e. E2-5, E2-6, E2-14, E3-4, E3-7, E3-8) and of *O. europaea maroccana* (M1-1, M1-2, M1-7).

### Nematode extraction, identification and quantification

All of the nematode analyses were performed in the nematode quarantine area (French Government Agreement No 80622) of the Research Unit, “Centre de Biologie pour la Gestion des Populations” (Montpellier, France).

A 250-cm^3^ wet aliquot was taken from each soil sample for nematode extraction using the elutriation procedure [23]. PPN belonging to the Aphelenchida, Dorylaimida, Triplonchida and Tylenchida orders were enumerated in 5-cm^3^ counting chambers [24] and identified at the genus level based on dichotomous keys [25] and at the species level with genus-specific keys. The population levels were expressed per dm^3^ of fresh soil. Concerning specific identification, the nematode suspensions were preserved in mixture of formalin and glycerine [26], and then adult specimens were processed according to Seinhorst method [27] and mounted onto slides [28] for microscopic observation. Root-knot nematodes (*Meloidogyne* spp.) were identified at the species level by biochemical (esterase patterns) and molecular (SCAR markers and 28S rDNA D2-D3 expansion segments) approaches [29].

### Analyses of nematode diversity

Several ecological indices were used:

- Taxonomical diversity: (i) the total number of PPN in a community (*N*); (ii) the species richness (*S*); (*iii*) the Shannon–Wiener diversity index *H’* (*H′* = −∑p_*i*_ *ln*p_*i*_, where p_*i*_ is the proportion of individuals in each species (iii) that quantifies the local diversity or the heterogeneity of diversity (*H′* ranges from 0 to *ln(S)*); and (iv) the evenness (*E* = *H′*/*ln S*) that quantifies the regularity of species distribution within the community (*E* varies between 0 and 1).
- Functional diversity: PPN species detected in communities were distributed into life-strategy groups according to the colonizer/persister value (*cp*-value) of the family to which they belong [30]. The diversity of the community was described by calculating: (i) the plant-parasitic index (PPI = ∑*cp*_*i*_n_*i*_/N), which quantifies the plant-feeding diversity of the communities; (ii) the relative mean abundance (%) of each *cp*-value class in a community calculated as follows: *Rcp*_*i*_ = *cp*_*i*_n_*i*_/N; (iii) the genus richness included in each *cp*-value class. PPN species were also assigned to the trophic groups according to their feeding habits [31, 32]: obligate plant feeders (OPF), facultative plant feeders (FPF) that alternatively feed on fungi, and fungal feeders (FF) that alternatively feed on plants. These trophic groups were also described according to (i) the relative mean abundance (%) of individuals within each of them, and (ii) the genus richness included in each [33].
- The structure of PPN communities was designed at the genus level. The dominance of each nematode genus in the samples was first estimated by modeling the abundance (A) and the frequency (F) of each genus in the whole samples [34]. Afterwards, PPN community structures were described according to multivariate statistical analyses.

### Data analyses

These diversity indices were calculated using the Vegan library [35]. In order to evaluate the impact of anthropogenic changes on biodiversity and community structures, different olive variables were defined according to olive-growing modalities: wild (WO), feral (FO), traditional or low-density cultivation (TR) and modern or high-density cultivation (HD), and according to olive irrigation conditions: irrigated or rainfed. The mean values of the different nematode diversity indices were compared according to olive propagation (wild vs cultivated) and to intensification practices (traditional vs high-density, irrigated vs rainfed). Principal Component Analysis (PCA) was carried out on nematode genera data in order to describe PPN community structures. To assess the impact of olive anthropogenic changes on taxonomical structures, a co-Inertia Analysis (CIA) was applied between olive-growing modality data (WO-FO-TR-HD) and PPN genera. The scarcest genera (with total abundance less than 1%) were then excluded from the dataset prior to running the analysis. These different multivariate analyses and graphs were performed using *ade4* library [36, 37]. All analyses were done using R version 3.3.2 [38]. The Wilcox (non-parametric) test was used for all pair-wise multiple comparisons. Differences obtained at levels of *P* < 0.05 were considered to be significant.

## Results

### PPN diversity associated with olive trees in Morocco

The PPN communities associated with olive trees in Morocco were highly diversified. A total of 117 species and 47 genera were identified. They belong to two families of Aphelenchida, to a family of Dorylaimida, to a family of Triplonchida and to 14 families of Tylenchida (Table 2).

**Table 2:** Plant-parasitic nematode taxa associated with olive trees in Morocco

Orders and families (*cp* value)	Species (trophic group)	Authors	Geographic regions														
			Rif							Jel		Kandar	Guerouane			Zaïane	
			Tanger	Fnideq	Tetouan	Asilah	Chefchaouen	Bni Harchen	Ouazzane	Taza	Guercif	Sefrou	El Hajeb	Meknes	Khemisset	Oulmes	Oued Zem
Aphelenchida																	
Aphelenchidae (2)	*Aphelenchus avenae* (F)	Bastian, 1865		+	+	+	+		+					+			
	*A. isomerus* (F)	Anderson and Hooper, 1980		+													
Aphelenchoididae (2)	*Aphelenchoides graminis* (F)	Baranovskaya and Haque, 1968		+													
	*A. helicus* (F)	Heyns, 1964															
	*A. saprophilus* (F)	Franklin, 1957		+		+											
	*Aprutides guidetti* (F)	Scognamiglio, 1974	+			+	+		+								
Dorylaimida																	
Longidoridae (5)	*Longidorus* sp. (OPF)	Micoletzky, 1922	+	+	+		+		+					+		+	
	*Xiphinema pachtaicum* (OPF)	Tulaganov, 1938	+		+	+	+	+	+								
	*X. turcicum* (OPF)	Luc and Dalmasso, 1964	+														
	*X. vuittenezi* (OPF)	Luc et al. 1964														+	+
	*Xiphinema* sp. (OPF)	Cobb, 1913		+	+	+	+	+	+			+		+			
Triplonchida																	
Trichodoridae (4)	*Paratrichodorus* sp. (OPF)	Siddiqi, 1974															
	*Trichodorus* sp. (OPF)	Cobb, 1913	+									+		+			
Tylenchida																	
Anguinidae (2)	*Ditylenchus emus* (FPF)	Khan et al., 1969															
	*D. equalis* (FPF)	Heyns, 1964				+			+								
	*Nothotylenchus acutus* (F)	Khan, 1965				+											
	*N. adasi* (F)	Syces, 1980						+									
	*N. geraerti* (F)	Kheiri, 1971	+				+		+								
	*N. medians* (F)	Thorne and Malek, 1968							+								
Criconematidae (3)	*Ogma rhombosquamatus* (OPF)	Mehta and Raski, 1981	+														
	*Criconema* sp. (OPF)	Hofmänner and Menzel, 1914															
	*Criconemella* sp. (OPF)	De Grisse and Loof, 1965												+			
	*Macroposthonia* sp. (OPF)	De Man, 1880					+										
Dolichoridae (3)	*Neodolichorhynchus microphasmis* (OPF)	Loof, 1960															
Heteroderidae (3)	*Heterodera riparia* (OPF)	Subbotin et al, 1997															
	*Heterodera* sp. (OPF)	Schmidt, 1871	+			+	+									+	+
Hoplolaimidae (3)	*Helicotylenchus canadensis* (OPF)	Waseem, 1961		+	+				+								
	*H. crassatus* (OPF)	Anderson, 1973	+	+	+	+	+	+	+				+	+	+	+	+
	*H. crenacauda* (OPF)	Sher, 1966	+														
	*H. digonicus* (OPF)	Perry, 1959	+	+		+	+		+							+	+
	*H. dihystera* (OPF)	Cobb, 1893	+	+	+	+	+	+	+						+		
	*H. exallus* (OPF)	Sher, 1966													+		
	*H. minzi* (OPF)	Sher, 1966													+		
	*H. pseudorobustus* (OPF)	Steiner, 1914															
	*H. tunisiensis* (OPF)	Siddiqi, 1964							+								
	*H. varicaudatus* (OPF)	Yuen, 1964	+	+	+	+		+	+							+	+
	*H. vulgaris* (OPF)	Yuen, 1964		+		+	+		+								
	*Helicotylenchus* sp. (OPF)	Steiner, 1945			+	+	+		+	+	+		+	+			
	*Rotylenchus buxophilus* (OPF)	Golden, 1956															
	*R. goodeyi* (OPF)	Loof and Oostenbrink, 1958															
	*R. pumilus* (OPF)	Perry, 1959	+	+		+			+						+		
	*R. robustus* (OPF)	de Man, 1876				+			+								
	*Rotylenchus* sp. (OPF)	Filipjev, 1936	+		+		+	+	+	+	+	+	+	+	+		+
Meloidogynidae (3)	*Meloidogyne arenaria* (OPF)	Neal, 1889	+														
	*M. hapla* (OPF)	Chitwood, 1949	+														
	*M. spartelensis* (OPF)	Ali et al. 2015	+														
	*Meloidogyne* sp2 (OPF)	Goeldi, 1892							+								
Paratylenchidae (2)	*Cacopaurus* sp. (OPF)	Thorne, 1943							+								
	*Paratylenchus* (*Gracilacus)* sp. (OPF)	Raski, 1962	+	+		+	+		+							+	
	*Paratylenchus* (*P.*) *microdorus* (OPF)	Andrássy, 1959							+								
	*P.* (*P.*) *nanus* (OPF)	Cobb, 1923	+														
	*P.* (*P.*) *sheri* (OPF)	Raski, 1973				+			+								
	*P.* (*G.*) *straeleni* (OPF)	de Coninck, 1931	+				+										
	*P.* (*P.*) *vandenbrandei* (OPF)	De Grisse, 1962	+				+										
	*P.* (*P.*) *veruculatus* (OPF)	Wu, 1962															
	*Paratylenchus* (*Paratylenchus*) sp. (OPF)	Micoletzky, 1922	+	+			+	+	+	+		+	+	+	+	+	+
Pratylenchidae (3)	*Pratylenchoides hispaniensis* (OPF)	Troccoli et al., 1997	+						+								
	*P. laticauda* (OPF)	Braun and Loof, 1967				+											
	*Pratylenchoides* sp. (OPF)	Winslow, 1958		+						+	+	+		+	+	+	+
	*Pratylenchus crenatus* (OPF)	Loof, 1960		+		+											
	*P. mediterraneus* (OPF)	Corbett, 1983							+								
	*P. neglectus* (OPF)	Rensch, 1924		+								+					
	*P. pinguicaudatus* (OPF)	Corbett, 1969				+											
	*P. thornei* (OPF)	Sher and Allen, 1953		+													
	*Pratylenchus* sp. (OPF)	Filipjev, 1936	+	+	+		+	+		+	+	+	+	+	+	+	
	*Zygotylenchus guevarai* (OPF)	Tobar Jiménez, 1963	+	+	+	+	+		+			+	+	+		+	
Psilenchidae (2)	*Psilenchus aestuarius* (FPF)	Andrássy, 1962															
	*P. hilarulus* (FPF)	de Man, 1921			+												
Rotylenchulidae (3)	*Rotylenchulus* sp. (OPF)	Linford and Oliveira, 1940	+			+	+		+	+	+	+				+	+
Telotylenchidae (3)	*Amplimerlinius globigerus* (OPF)	Siddiqi, 1979				+	+	+	+					+	+		
	*A. intermedius* (OPF)	Bravo, 1976							+								
	*A. paraglobigerus* (OPF)	Castillo et al., 1990															
	*Bitylenchus aerolatus* (OPF)	Tobar Jiménez, 1970															
	*Merlinius brevidens* (OPF)	Allen, 1955	+	+	+	+	+		+					+	+	+	+
	*M. microdorus* (OPF)	Geraert, 1966						+									
	*M. nothus* (OPF)	Allen, 1955	+	+			+	+									
	*Merlinius* sp. (OPF)	Siddiqi, 1970								+		+					
	*Nagelus obscurus* (OPF)	Allen, 1955		+													
	*Paratrophurus loofi* (OPF)	Arias, 1970				+											
	*Scutylenchus lenorus* (OPF)	Brown, 1956															
	*S. mamillatus* (OPF)	Tobar-Jiménez, 1966															
	*S. tessellatus* (OPF)	Goodey, 1952															
	*Telotylenchus avaricus* (OPF)	Kleynhans, 1975															
	*T. paaloofi* (OPF)	Tikyani and Khera, 1970															
	*T. ventralis* (OPF)	Loof, 1963							+								
	*Trophurus sculptus* (OPF)	Loof, 1956		+		+	+										
	*Tylenchorhynchus clarus* (OPF)	Allen, 1955	+		+	+		+	+								
	*T. crassicaudatus* (OPF)	Williams, 1960						+									
	*Tylenchorhynchus* sp. (OPF)	Cobb, 1913			+					+	+	+	+	+			+
Tylenchidae (2)	*Aglenchus agricola* (FPF)	de Man, 1884						+									
	*Basiria flandriensis* (FPF)	Gerraert, 1968				+											
	*B. graminophila* (FPF)	Siddiqi, 1959	+			+	+								+		
	*B. tumida* (FPF)	Colbran, 1960	+					+									
	*Boleodorus clavicaudatus* (F)	Thorne, 1941					+										
	*B. thylactus* (F)	Thorne, 1941		+		+	+										
	*B. volutus* (F)	Lima and Siddiqi, 1963				+											
	*Coslenchus gracilis* (FPF)	Andrássy, 1982						+									
	*Discotylenchus* sp. (FPF)	Siddiqi, 1980															
	*Filenchus andrassyi* (FPF)	Szczygieł, 1969														+	+
	*F. baloghi* (FPF)	Andrássy, 1958						+								+	+
	*F. filiformis* (FPF)	Bütschli, 1873	+	+	+	+	+	+	+				+	+	+		
	*F. hamatus* (FPF)	Thorne and Malek, 1968		+													
	*F. misellus* (FPF)	Andrássy, 1958		+		+	+								+	+	+
	*F. sandneri* (FPF)	Wasilewska, 1965	+														
	*Filenchus* sp. (FPF)	Andrássy, 1954			+		+	+	+	+	+	+	+	+			
	*Irantylenchus vicinus* (FPF)	Szczygieł, 1970				+											
	*Malenchus acarayensis* (FPF)	Andrássy, 1968															
	*M. andrassyi* (FPF)	Merny, 1970															
	*M. exiguus* (FPF)	Massey, 1969					+										
	*Malenchus* sp. (FPF)	Andrássy, 1968															
	*Miculenchus salvus* (FPF)	Andrássy, 1959															
	*Ottolenchus discrepans* (FPF)	Andrássy, 1954															
	*O. facultativus* (FPF)	Szczygieł, 1970				+									+		
	*Tylenchus elegans* (FPF)	De Man, 1876				+											
	*Tylenchus* sp. (FPF)	Bastian, 1865															

Orders and families (*cp* value)	Species (trophic group)	Authors	Geographic regions														
			Tadla		Haouz						Souss						
			Beni Mellal	El Ksiba	El Kelaa Des Sraghna	Tamellalt	Sidi Bou Othmane	Marrakech	Tahnaout	Asni	Tiguert	Aourir	Ouled Teima	Taroudant	Ouled Berhil	Aoulouz	
Aphelenchida																	
Aphelenchidae (2)	*Aphelenchus avenae* (F)	Bastian, 1865	+	+				+			+		+	+		+	
	*A. isomerus* (F)	Anderson and Hooper, 1980															
Aphelenchoididae (2)	*Aphelenchoides graminis* (F)	Baranovskaya and Haque, 1968															
	*A. helicus* (F)	Heyns, 1964						+									
	*A. saprophilus* (F)	Franklin, 1957		+							+						
	*Aprutides guidetti* (F)	Scognamiglio, 1974															
Dorylaimida																	
Longidoridae (5)	*Longidorus* sp. (OPF)	Micoletzky, 1922	+	+							+		+				
	*Xiphinema pachtaicum* (OPF)	Tulaganov, 1938									+						
	*X. turcicum* (OPF)	Luc and Dalmasso, 1964															
	*X. vuittenezi* (OPF)	Luc et al. 1964															
	*Xiphinema* sp. (OPF)	Cobb, 1913	+	+	+					+	+	+	+	+	+		
Triplonchida																	
Trichodoridae (4)	*Paratrichodorus* sp. (OPF)	Siddiqi, 1974						+									
	*Trichodorus* sp. (OPF)	Cobb, 1913		+													
Tylenchida																	
Anguinidae (2)	*Ditylenchus emus* (FPF)	Khan et al. 1969									+						
	*D. equalis* (FPF)	Heyns, 1964									+						
	*Nothotylenchus acutus* (F)	Khan, 1965															
	*N. adasi* (F)	Syces, 1980															
	*N. geraerti* (F)	Kheiri, 1971	+								+		+				
	*N. medians* (F)	Thorne and Malek, 1968															
Criconematidae (3)	*Ogma rhombosquamatus* (OPF)	Mehta and Raski, 1981															
	*Criconema* sp. (OPF)	Hofmänner and Menzel, 1914			+												
	*Criconemella* sp. (OPF)	De Grisse and Loof, 1965															
	*Macroposthonia* sp. (OPF)	De Man, 1880															
Dolichoridae (3)	*Neodolichorhynchus microphasmis* (OPF)	Loof, 1960											+				
Heteroderidae (3)	*Heterodera riparia* (OPF)	Subbotin et al., 1997															
	*Heterodera* sp. (OPF)	Schmidt, 1871		+	+						+		+	+			
Hoplolaimidae (3)	*Helicotylenchus canadensis* (OPF)	Waseem, 1961								+							
	*H. crassatus* (OPF)	Anderson, 1973	+	+	+			+	+	+	+		+	+		+	
	*H. crenacauda* (OPF)	Sher, 1966	+		+			+									
	*H. digonicus* (OPF)	Perry, 1959	+	+								+					
	*H. dihystera* (OPF)	Cobb, 1893		+	+	+	+	+		+		+	+				
	*H. exallus* (OPF)	Sher, 1966															
	*H. minzi* (OPF)	Sher, 1966															
	*H. pseudorobustus* (OPF)	Steiner, 1914					+	+									
	*H. tunisiensis* (OPF)	Siddiqi, 1964															
	*H. varicaudatus* (OPF)	Yuen, 1964		+				+		+		+				+	
	*H. vulgaris* (OPF)	Yuen, 1964	+		+			+								+	
	*Helicotylenchus* sp. (OPF)	Steiner, 1945		+	+	+		+	+		+		+	+	+		
	*Rotylenchus buxophilus* (OPF)	Golden, 1956						+									
	*R. goodeyi* (OPF)	Loof and Oostenbrink, 1958									+						
	*R. pumilus* (OPF)	Perry, 1959		+				+									
	*R. robustus* (OPF)	de Man, 1876															
	*Rotylenchus* sp. (OPF)	Filipjev, 1936	+	+	+			+	+	+	+	+	+	+	+	+	
Meloidogynidae (3)	*Meloidogyne arenaria* (OPF)	Neal, 1889															
	*M. hapla* (OPF)	Chitwood, 1949															
	*M. spartelensis* (OPF)	Ali et al, 2015															
	*Meloidogyne* sp2 (OPF)	Goeldi, 1892			+			+			+						
Paratylenchidae (2)	*Cacopaurus* sp. (OPF)	Thorne, 1943															
	*Paratylenchus* (*Gracilacus)* sp. (OPF)	Raski, 1962										+		+			
	*Paratylenchus* (*P.*) *microdorus* (OPF)	Andrássy, 1959									+						
	*P.* (*P.*) *nanus* (OPF)	Cobb, 1923															
	*P.* (*P.*) *sheri* (OPF)	Raski, 1973															
	*P.* (*G.*) *straeleni* (OPF)	de Coninck, 1931															
	*P.* (*P.*) *vandenbrandei* (OPF)	De Grisse, 1962															
	*P.* (*P.*) *veruculatus* (OPF)	Wu, 1962						+									
	*Paratylenchus* (*Paratylenchus*)sp. (OPF)	Micoletzky, 1922	+	+	+	+	+	+	+	+	+	+	+	+	+	+	
Pratylenchidae (3)	*Pratylenchoides hispaniensis* (OPF)	Troccoli et al. 1997												+			
	*P. laticauda* (OPF)	Braun and Loof, 1967															
	*Pratylenchoides* sp. (OPF)	Winslow, 1958	+	+				+			+	+	+	+			
	*Pratylenchus crenatus* (OPF)	Loof, 1960															
	*P. mediterraneus* (OPF)	Corbett, 1983															
	*P. neglectus* (OPF)	Rensch, 1924		+													
	*P. pinguicaudatus* (OPF)	Corbett, 1969	+					+									
	*P. thornei* (OPF)	Sher and Allen, 1953															
	*Pratylenchus* sp. (OPF)	Filipjev, 1936	+	+	+		+	+	+	+	+	+	+			+	
	*Zygotylenchus guevarai* (OPF)	Tobar Jiménez, 1963			+						+			+			
Psilenchidae (2)	*Psilenchus aestuarius* (FPF)	Andrássy, 1962			+												
	*P. hilarulus* (FPF)	de Man, 1921															
Rotylenchulidae (3)	*Rotylenchulus* sp. (OPF)	Linford and Oliveira, 1940	+	+	+			+			+	+	+	+			
Telotylenchidae (3)	*Amplimerlinius globigerus* (OPF)	Siddiqi, 1979						+									
	*A. intermedius* (OPF)	Bravo, 1976															
	*A. paraglobigerus* (OPF)	Castillo et al. 1990															
	*Bitylenchus aerolatus* (OPF)	Tobar Jiménez, 1970						+									
	*Merlinius brevidens* (OPF)	Allen, 1955	+		+		+	+		+	+		+	+		+	
	*M. microdorus* (OPF)	Geraert, 1966					+										
	*M. nothus* (OPF)	Allen, 1955															
	*Merlinius* sp. (OPF)	Siddiqi, 1970		+	+			+					+				
	*Nagelus obscurus* (OPF)	Allen, 1955		+													
	*Paratrophurus loofi* (OPF)	Arias, 1970															
	*Scutylenchus lenorus* (OPF)	Brown, 1956								+		+	+				
	*S. mamillatus* (OPF)	Tobar-Jiménez, 1966									+						
	*S. tessellatus* (OPF)	Goodey, 1952								+							
	*Telotylenchus avaricus* (OPF)	Kleynhans, 1975											+				
	*T. paaloofi* (OPF)	Tikyani and Khera, 1970	+							+			+				
	*T. ventralis* (OPF)	Loof, 1963						+			+		+				
	*Trophurus sculptus* (OPF)	Loof, 1956															
	*Tylenchorhynchus clarus* (OPF)	Allen, 1955			+	+	+	+	+		+	+	+		+		
	*T. crassicaudatus* (OPF)	Williams, 1960										+					
	*Tylenchorhynchus* sp. (OPF)	Cobb, 1913	+	+	+	+		+	+		+		+	+	+		
Tylenchidae (2)	*Aglenchus agricola* (FPF)	de Man, 1884															
	*Basiria flandriensis* (FPF)	Gerraert, 1968															
	*B. graminophila* (FPF)	Siddiqi, 1959														+	
	*B. tumida* (FPF)	Colbran, 1960	+								+						
	*Boleodorus clavicaudatus* (F)	Thorne, 1941															
	*B. thylactus* (F)	Thorne, 1941	+	+				+			+		+			+	
	*B. volutus* (F)	Lima and Siddiqi, 1963															
	*Coslenchus gracilis* (FPF)	Andrássy, 1982															
	*Discotylenchus* sp. (FPF)	Siddiqi, 1980	+														
	*Filenchus andrassyi* (FPF)	Szczygieł, 1969															
	*F. baloghi* (FPF)	Andrássy, 1958															
	*F. filiformis* (FPF)	Bütschli, 1873	+	+	+	+	+	+	+	+	+					+	
	*F. hamatus* (FPF)	Thorne and Malek, 1968			+	+		+	+	+						+	
	*F. misellus* (FPF)	Andrássy, 1958									+					+	
	*F. sandneri* (FPF)	Wasilewska, 1965									+						
	*Filenchus* sp. (FPF)	Andrássy, 1954	+	+	+	+		+	+	+	+	+	+	+	+	+	
	*Irantylenchus vicinus* (FPF)	Szczygieł, 1970			+												
	*Malenchus acarayensis* (FPF)	Andrássy, 1968									+						
	*M. andrassyi* (FPF)	Merny, 1970														+	
	*M. exiguus* (FPF)	Massey, 1969															
	*Malenchus* sp. (FPF)	Andrássy, 1968														+	
	*Miculenchus salvus* (FPF)	Andrássy, 1959						+									
	*Ottolenchus discrepans* (FPF)	Andrássy, 1954						+									
	*O. facultativus* (FPF)	Szczygieł, 1970															
	*Tylenchus elegans* (FPF)	De Man, 1876															
	*Tylenchus* sp. (FPF)	Bastian, 1865			+					+		+					

Trophic groups: *FF* fungal feeders, *FPF* facultative plant feeders, *OPF* obligate plant feeders

At the family level, the Tylenchidae and Telotylenchidae were dispersed in all the regions sampled; they were the most diversified families, including 11, 9 genera in each, respectively. However, each genus was often represented by one or two species only (e.g. *Amplimerlinus*, *Bitylenchus*, *Tylenchus*). Most of these species were very rare as they were detected in one or two sites only (e.g. *Aglenchus agricola*, *Coslenchus gracilis* and *Paratrophurus loofi* in the Rif region). In contrast, the Hoplolaimidae family was represented by two genera only (*Helicotylenchus* and *Rotylenchus*), but the number of species identified in each genus was high (11 and 4 species, respectively), and they were distributed in all the regions, except in eastern Morocco (the Kandar and Jel regions). Longidoridae and Trichodoridae nematodes were detected mostly in the Rif region. Root-lesion nematodes (e.g. *Pratylenchus*) and Pin nematodes Paratylenchidae (e.g. *Paratylenchus*) were dispersed at all the sites surveyed. Four root-knot nematodes species were identified: *Meloidogyne arenaria* and *M. hapla* were detected in the Rif region, *M. javanica* was generally detected in southern Morocco (in the Souss and Haouz regions) and in the Guerouane and Tadla regions. *M. spartelensis* is a new species identified in the Rif region; another new species seems to occur in the Souss region (identification is in progress). Other families such as Criconematidae and Psilenchidae were detected in a few sites.

Among the 47 identified genera, *Filenchus*, *Helicotylenchus*, *Merlinius*, *Paratylenchus*, *Pratylenchus*, *Rotylenchus*, *Tylenchorhynchus* and *Xiphinema* were the most widespread in olive soils. Considering the species level, 11 *Helicotylenchus* species (Hoplolaimidae) were frequently collected in olive samples. Among them, *H. crassatus* was clearly the most dominant species (occurring in 58% of the samples). It was present in all regions except in the Jel and Kandar regions. *H. dihystera* and *H. varicaudatus* also occurred in 43 and 32% of the samples, respectively. In contrast, *H. exallus* and *H. minzi*, detected in the Guerouane region, and *H. pseudorobustus*, detected in the Haouz region, were scarcer. In addition, *Merlinius brevidens* (Telotylenchidae) and *Filenchus filiformis* (Tylenchidae) were also frequently recovered (51 and 40% of the samples, respectively).

### Diversity of PPN communities according to anthropogenic changes

Diversity indices mean values were compared between to the four olive-growing modalities and between rainfed and irrigated olive samples.

#### (a) Taxonomical diversity

The total number of PPN (*N*) was up to two times higher on cultivated (HD & TR) than on non-cultivated olive (WO & FO). Similarly on irrigated olive, the total number of PPN was higher (Table 3). In contrast, the PPN communities were significantly richer in species (*S*), more diversified (*H′*) and more homogenously distributed (*E*) in communities on WO and FO and on rainfed olive than on TR and HD and on irrigated olive.

**Table 3:** Taxonomical diversity indices in PPN communities associated with olive (mean values) according to olive-growing modalities and water supply

Olive variables	Nb of samples	N	S	Hʹ	E
Growing modality					
WO	88	2227 *b*	10.31 *a*	1.55 *a*	0.68 *a*
FO	75	2751 *b*	9.51 *a*	1.58 *a*	0.69 *a*
TR	40	4369 *a*	7.50 *b*	1.24 *b*	0.58 *a*
HD	10	4352 *a*	6.90 *b*	1.04 *b*	0.50 *b*
Water supply					
Rainfed	171	2512 *b*	9.87 *a*	1.56 *a*	0.69 *a*
Irrigated	42	4365 *a*	7.36 *b*	1.19 *b*	0.56 *b*

The letters (*a*–*c*) indicate significant differences among the variables measured according to ANOVA and Wilcoxon tests. *P* < 0.05

*WO* wild olive, *FO* feral olive, *TR* traditional cultivation, *HD* high-density cultivation, *N* total number of PPN/dm^3^ of soil, *S* species richness, *H′* local diversity, *E* evenness

#### (b) Functional diversity

The PPN identified were allocated in all the parasitic *cp*-values (*cp*-2 to *cp*-5 groups, Table 2). The WO and HD modalities revealed nematode communities with significantly higher plant-parasitic indices (PPI) than those in FO and in TR orchards (Table 4). This means that WO and HD olive areas had significantly more plant-feeding nematodes with higher *cp* values than other olive systems. The most opportunist/colonizer PPN (*cp*-2 and *cp*-3) dominated in all the communities (44 and 48%, respectively; Table 2). The overall abundance and occurrence of the persister nematodes (*cp*-4 and *cp*-5) was very low (4% for each *cp* class). Any effect was recorded on the *cp*-4 class. *Cp*-2 and *cp*-3 nematodes were more abundant in TR and HD, while *cp*-5 nematodes occurred more often in WO areas and were completely absent in HD orchards.

**Table 4:** Functional diversity in PPN communities on olive (mean values) according to olive-growing modalities and water supply

Olive variables	PPI	R*cp*-2	R*cp*-3	R*cp*-4	R*cp*-5	FF	FPF	OPF
Growing modality								
WO	2.65 *a*	45.58 *a*	48.89 *b*	0.08	5.45 *a*	8.69 *a*	32.63 *b*	58.68 *ab*
FO	2.57 *ab*	46.19 *a*	52.19 *b*	0.03	1.59 *b*	3.62 *b*	39.35 *ab*	57.03 *ab*
TR	2.49 *b*	52.62 *a*	46.68 *b*	0.04	0.66 *b*	3.69 *b*	46.89 *a*	49.42 *b*
HD	2.74 *a*	25.96 *b*	73.71 *a*	0.33	0.00 *b*	0.12 *b*	25.13 *b*	74.76 *a*
Water supply								
Rainfed	2.61	45.91	50.69	0.05	3.35 *a*	5.93	36.28	57.78
Irrigated	2.55	46.27	53.11	0.11	0.50 *b*	2.84	41.71	55.45

The letters (a–c) indicate significant differences among the variables measured according to ANOVA and Wilcoxon tests. *P* < 0.05

*WO* wild olive, *FO* feral olive, *TR* traditional cultivation, *HD* high-density cultivation, *PPI* plant parasitic index, relative mean abundance (%) of each *cp*-value (R*cp*-i) and of each trophic group (*FF* fungal feeders, *FPF* facultative plant feeders, *OPF* obligate plant feeders)

Concerning the trophic groups within communities, the OPF nematodes were the most dominant (62%), while the FPF and the FF nematodes were the least frequent (26 and 12%, respectively). FF nematodes were significantly more numerous in WO areas (Table 4). FPF and OPF nematodes were more abundant in TR and HD orchards, respectively. The ratio between FPF and OPF nematodes was unbalanced in favor of OPF in HD orchards, and in favor of FPF in TR orchards and in FO areas. The rainfed-irrigation modalities did not have any effect on the trophic groups.

The *cp*-2, *cp*-3, FPF and OPF functional groups were represented by the highest number of genera (44, 48, 26 and 62%, respectively). Comparing this richness in each group between olive-growing modalities only, the PPN communities detected in WO and FO demonstrated higher richness and diversity compared to those detected in TR and HD (Table 5).

**Table 5:** Genus richness of PPN within each functional group according to olive-growing modalities

Olive-growing modalities	*cp*-2	*cp*-3	*cp*-4	*cp*-5	FF	FPF	OPF
WO	24	23	1	3	5	14	32
FO	13	14	1	2	3	8	19
TR	11	11	1	2	4	6	15
HD	5	11	2	0	1	3	14

*WO* wild olive, *FO* feral olive, *TR* traditional cultivation, *HD* high-density cultivation, *cp*-2 to *cp*-5 *cp*-values, *FF* fungal feeders, *FPF* facultative plant feeders, *OPF* obligate plant feeders

#### (c) Community patterns

**Fig. 2:**
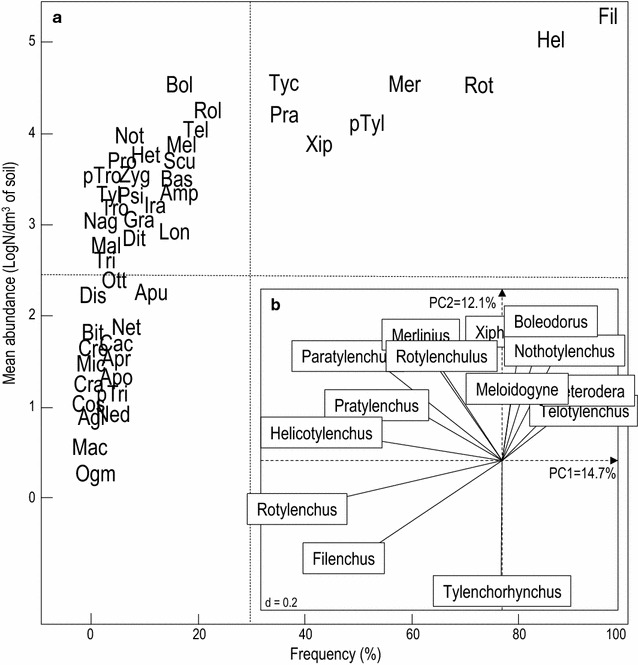
Plant-parasitic nematode communities in the olive areas surveyed in Morocco. **a** Dominance diagram of the nematode genera. Codes for nematode genera are given in Table 6. *Dotted lines* indicate delineation between low and high abundances and frequencies as described in [34]. **b** Plant-parasitic nematode community patterns (PCA loading plot for the nematode genera)

Community structure was described at the genus level. Modeling the dominance of each genus in the samples (Fig. 2a), 83% of the genera were classified as less frequent (F < 30%) according to the model and 35% as occasional (F < 5%). A total of 62.5% of the nematode genera were classified as highly abundant according to the abundance threshold defined by the model (A = 200 nematodes/dm^3^ of soil). Eight genera were classified as dominant (F ≥ 30% and A ≥ 10,000 nematodes/dm^3^ of soil): *Filenchus* and *Helicotylenchus* (F > 80%); and *Rotylenchus*, *Merlinius*, *Paratylenchus*, *Xiphinema*, *Pratylenchus* and *Tylenchorhynchus* (40 < F < 70%). Six other highly abundant genera were less frequent, including root-knot nematodes (*Meloidogyne* spp., F = 12.2%) and cyst nematodes (*Heterodera* spp., F = 10%). No genus was found to be frequent and in low abundance.

**Table 6:** Nematodes genera and their corresponding codes

PPN genus	Code	PPN genus	Code	PPN genus	Code
*Aglenchus*	Agl	*Helicotylenchus*	Hel	*Paratrophorus*	pTro
*Amplimerlinius*	Amp	*Heterodera*	Het	*Paratylenchus*	pTyl
*Aphelenchoides*	Apo	*Irantylenchus*	Ira	*Pratylenchoides*	Pro
*Aphelenchus*	Apu	*Longidorus*	Lon	*Pratylenchus*	Pra
*Aprutides*	Apr	*Macroposthenia*	Mac	*Psilenchus*	Psi
*Basiria*	Bas	*Malenchus*	Mal	*Rotylenchulus*	Rol
*Bitylenchus*	Bit	*Meloidogyne*	Mel	*Rotylenchus*	Rot
*Boleodorus*	Bol	*Merlinius*	Mer	*Scutylenchus*	Scu
*Cacopaurus*	Cac	*Miculenchus*	Mic	*Telotylenchus*	Tel
*Coslenchus*	Cos	*Nagelus*	Nag	*Trichodorus*	Tri
*Criconema*	Cra	*Neodolichorhynchus*	Ned	*Trophurus*	Tro
*Criconemella*	Cre	*Neotylenchus*	Net	*Tylenchorhynchus*	Tyc
*Discotylenchus*	Dis	*Nothotylenchus*	Not	*Tylenchus*	Tyl
*Ditylenchus*	Dit	*Ogma*	Ogm	*Xiphinema*	Xip
*Filenchus*	Fil	*Ottolenchus*	Ott	*Zygotylenchus*	Zyg
*Gracilacus*	Gra	*Paratrichodorus*	pTri		

As shown by the PCA loading plot of the nematode taxa (Fig. 2b), Hoplolaimidae nematodes (*Helicotylenchus* and *Rotylenchus*), and *Paratylenchus*, *Filenchus* and *Pratylenchus* genera to a lesser extent, were correlated to the PC1 axis (negative values). The PC2 axis indicated contrasted positions for *Tylenchorhynchus* spp. (negative values), opposed to *Boleodorus*, *Xiphinema*, *Nothotylenchus*, *Merlinius*, *Rotylenchulus*, *Meloidogyne*, *Heterodera* and *Telotylenchus* (positive values).

### Correspondences between PPN community patterns and olive-growing modalities

**Fig. 3:**
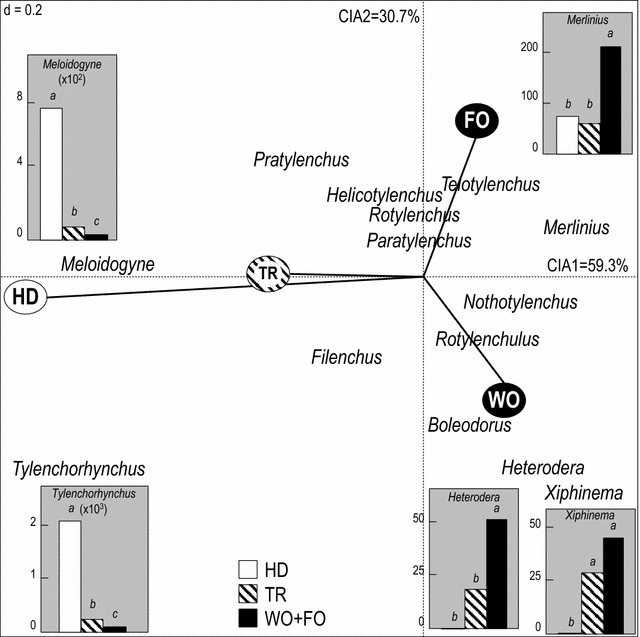
CIA loading plot for the nematode genera and the olive modalities. Histograms represent the mean comparisons of nematode abundances between olive-growing modality groups arranged according to their CIA1 eigenvalues. *WO* wild olive, *FO* feral olive, *TR* traditional cultivation, *HD* high-density cultivation

Considering olive-growing modalities, the loading plot of the Co-Inertia Analysis (CIA) analysis between nematode and olive data (Fig. 3) indicated an important contribution of the anthropogenic gradient (WO-FO-TR-HD) to the CIA1 axis. The CIA2 axis was essentially correlated with the feral growing modality (FO, positive values) and with the wild olive (WO, negative values). Regarding the projection of the nematode genera in the loading plot (Fig. 3), the analysis indicated that the genera *Merlinius*, *Xiphinema*, *Heterodera*, *Nothotylenchus, Rotylenchulus* and *Boleodorus* were correlated with WO. In contrast, *Meloidogyne* and *Tylenchorhynchus* were enhanced by cultivation practices (especially HD). The other nematode genera (*Filenchus, Pratylenchus*) were more closely related to TR, while *Telotylenchus, Helicotylenchus*, *Rotylenchus* and *Paratylenchus* were more closely related to FO. The mean comparisons of nematode abundances between the modality groups arranged according to their CIA1 eigenvalues (HD, TR and WO + FO) (Fig. 3) confirmed that *Meloidogyne* and *Tylenchorhynchus* nematodes were significantly more abundant in HD orchards compared to TR orchards or to WO + FO. Some significant differences were also detected between traditional and non-cultivated olive orchards. However, other nematodes such as *Merlinius*, *Xiphinema* and *Heterodera* were found to be significantly more abundant in WO + FO compared to cultivated olive conditions (HD, TR).

## Discussion

Biodiversity is an essential ecological phenomenon because it represents a complex set of interacting ecological, evolutionary, biogeographical and physical processes [39]. Native biodiversity is being lost at a rapid rate owing to anthropogenic causes, including habitat destruction, pollution and the spread of non-native species [1, 40]. In this context, the main focus of this study was to understand how human activities (e.g. agricultural practices) in ecosystems could impact the diversity of PPN communities. The Mediterranean olive tree is particularly suitable for this study because it concerns ancient ecosystems with post-glacial refugia [16], many spots of Oleaster and many cases of feral olive. It also offers a large range of varieties, cultivated traditionally or at high-density, as present in Morocco.

### PPN diversity associated with olive trees in Morocco

The PPN fauna and their distribution was totally unknown in Morocco before this study, except for a few reports on some nematodes such as root-knot nematodes *Meloidogyne morocciensis* [41] and cereal cyst nematodes [42]. This study clearly highlights a high taxonomical diversity of PPN communities where 117 species belonging to 47 genera were recorded. In addition, the study adds taxa (seven genera and 60 species) that were recorded for the first time in association with olive trees worldwide. The dominance pattern was also revealed by PCA analyses that demonstrated that the nematode dataset was mainly structured by the most frequent and abundant genera, and by less frequent but abundant nematodes to a lesser extent. The communities observed were mainly dominated by *Filenchus* and *Helicotylenchus* genera, and other nematodes such as *Rotylenchus*, *Merlinius*, *Paratylenchus*, *Xiphinema*, *Pratylenchus* and *Tylenchorhynchus.* Some of them have been previously reported as widespread on olive trees worldwide [15]. High population levels of some nematode genera such as root-knot nematodes (*Meloidogyne* spp.) and cyst nematodes (*Heterodera* spp.), considered as very dangerous soil-borne plant pests were also recorded [43].

The taxonomical diversity of PPN analyzed in Morocco is the greatest when compared to other surveys on olive trees that documented 223 species worldwide (reported in [14, 15, 44–47]). This high diversity and the detection of new taxa could be essentially explained by: (i) a large sampling effort (213 soil samples corresponding to 363 trees sampled), conducted along a long transect (about 900 km) covering a wide range of olive-growing regions in Morocco; and (ii) a large proportion of samples collected in wild and feral olive areas (163 samples). These olive habitats could be considered as reservoirs of high diversity where a part remains unknown [48]. As evidence, a new root-knot nematode species, *Meloidogyne spartelensis*, was detected on wild olive in Northern Morocco [49]. However, other species could not be detected because they may occur only under unidentifiable life stages (e.g. juveniles), or their development may be linked to other periods of the year or to specific microhabitats [50]. As an example, no *Rotylenchulus* could be identified at the species level because all individuals were in the juvenile stage.

### Impact of anthropogenic changes on the PPN communities associated with olive trees in Morocco

Taxonomical diversity indices were revealed impacted by olive propagation practices (from wild to cultivated olive): a high PPN richness was found in non-cultivated olive areas (wild and feral), with an equal distribution of species within communities (high evenness), contrary to what was observed in cultivated orchards (traditional and high-density). Nematode abundance was also significantly higher in orchards. A main conclusion also arose in this study that showed that PPN are abundant in cultivated conditions while richness, local diversity and evenness are low, and vice versa in non-cultivated conditions. In other words, a high PPN species diversity within a community may prevent the multiplication of the species as a potential effect of trade-off interactions between nematode species and/or between them and other soil microorganisms [51, 52].

The study also highlighted the impact of anthropogenic practices on the functional diversity in communities: persisters and fungal-feeders were more diverse and numerous in wild olive conditions, whereas colonizers were frequently present under high-density conditions. Colonizer nematodes were represented by fewer genera, confirming imbalance between the high relative abundance and the low-genus richness and vice versa. Moreover, *cp*-5 nematodes were particularly related to wild olive, and totally absent under high-density olive cultivation conditions. This is consistent with other studies that demonstrated that cultivation intensification usually does not reduce the number of nematode trophic groups, but may change the composition of these groups [53]. The taxonomical structures of the communities were also distinguished between wild and cultivated olive: genera such as *Xiphinema* and *Heterodera* were detected in relation to natural ecosystems (wild olive), while others (e.g. *Meloidogyne* and *Tylenchorhynchus*) were favoured in cultivated areas. Dominant taxa such as *Helicotylenchus*, *Rotylenchus* and *Filenchus* did not appear to be impacted, which could explain their high dominance in the samples.

The taxonomical biodiversity indices were affected by the intensification level of farming systems between low or high tree-density orchards. The genus richness was usually higher in traditional than in high-density orchards. However, the intensification practices also impacted the functional diversity, as abundant *cp*-2 and FPF nematodes were found in traditional orchards, while *cp*-3 and OPF were more abundant in high-density olive orchards. The taxonomical structures of communities were also affected by olive cultivation intensification: genera such as *Meloidogyne* spp. and *Tylenchorhynchus* spp. were dominant in high-density orchards, whereas the traditional orchards were more favorable for the development of other genera such as *Pratylenchus* spp.

This study suggests that the PPN communities associated with non-cultivated olives (wild and feral) are not disturbed as a consequence of low or no human intervention in these ecosystems. This is consistent with other ecological observations that show that lowly-disturbed ecosystems generally host more diverse communities of soil organisms, as demonstrated for earthworms [54], for PPN [55] and for other soil biota communities [5, 6]. That is completely reversed in cultivated areas where the PPN communities were characterized by high abundances and low PPN’s diversity. It is usually assumed that cropping systems are disturbed by human activities via agricultural practices (e.g. crop intensification, irrigation, tillage). These anthropogenic practices lead to species decline, as it has already been demonstrated on bees, birds and plants species [56], and soil biota [5, 6] including nematodes [57]. The decrease of nematodes diversity with increasing human activities can be attributed to several constraints such as physical disturbances, changes in quantity and quality of organic matter being returned to the soil and to the increase in the number of specific plant-feeding nematodes that are favoured by the selected crops [58].

These impacts on communities could be related to the biological characteristics of nematodes, leading them to respond differently to disturbances in their environment. These conditions induce favourable environments for PPN multiplication, especially irrigation, which enhances the development of roots [14]. This was consistent with others observations in southern Morocco [59]. This could explain the high abundance of colonizer species and, consequently, the high pathogenicity (PPI value) of the communities recorded in these cropping conditions. Moreover, agricultural practices applied in olive are very likely to select and multiply the most competitive and harmful PPN species such as *Meloidogyne* spp. in high-density orchards. That could also explain the absence of persister species in these conditions, since they are very sensitive to environmental disturbances. That agrees with previous studies [53] showing that the greater *cp*-value nematodes are usually associated with low stress and undisturbed environments [9].

## Conclusion

Anthropogenic changes such as propagation and intensification practices greatly impact the diversity of PPN communities associated with olive trees. Cultural practices (from wild to cultivated ecosystems or cropping intensification) could lead to community rearrangements in favour of highly pathogenic species defined as major agricultural pests [60]. In this vein, intensive production systems (high-yield varieties, irrigation, fertilization, etc.) induce environmental conditions suitable for the development of soil-borne diseases caused directly or indirectly (e.g. *Verticillium* wilt) by nematodes [14], such as root-knot (Meloidogynidae) and root-lesion (Pratylenchidae) nematodes. These groups of nematodes are known to affect olive production worldwide [15] and to be among the most frequent nematodes in nurseries [61]. Considering that the dispersal of PPN over long distances is passive (via contaminated irrigation, infected planting material or the dispersion of infested soil, etc. [30]), olive tree protection relies first on the use of healthy plant material (rootstocks) transplanted in a soil free of these parasites. The first step in avoiding PPN therefore starts in nurseries from where they could be introduced into olive orchards. This study also underlined PPN diversity and community structures as relevant indicators to assess resilient strategies in olive cropping systems. Further investigations should therefore focus on community rearrangements and on interactions between species co-existence mechanisms in order to develop diversity conservation or restoration (resilience) strategies [60] instead of reducing the most pathogenic species.

## Abbreviations

A: abundance
ade4: analyse de données écologiques version 4
ANOVA: analysis of variation
CIA: co-inertia analysis
*cp*-value: a functional diversity index assigned to families of soil nematodes, which are categorized into a 1-5 colonizer-persister series
*E*: evenness
E1, E2, E3, M1, M2, M7: olive chloroplast lines
E1-1, E2-1, E3-4, M1-1, etc.: olive chloroplast haplotypes
F: frequency
FF: fungal feeders
FO: feral olive
FPF: facultative plant feeders
H’: Shannon–Wiener diversity index
HD: high-density or modern cultivation
*ln*: natural logarithm
N: total number of nematodes in a community
OPF: obligate plant feeders
PCA: principal component analysis
p_*i*_: proportion of individuals in each species *i*
PPI: plant-parasitic index
PPN: plant-parasitic nematodes
*Rcp*: relative mean abundance (%) of each *cp*-value class in a community
S: species richness
SCAR: sequence characterized amplified region
TR: traditional or low-density cultivation
WO: wild olive
